# Environmental Obesogens and Their Impact on Susceptibility to Obesity: New Mechanisms and Chemicals

**DOI:** 10.1210/endocr/bqaa024

**Published:** 2020-02-18

**Authors:** Riann Jenay Egusquiza, Bruce Blumberg

**Affiliations:** 1 Department of Developmental and Cell Biology, University of California Irvine, Irvine, California; 2 Department of Pharmaceutical Sciences, University of California Irvine, Irvine, California; 3 Department of Biomedical Engineering, University of California Irvine, Irvine, California

**Keywords:** obesity, obesogen, endocrine disrupting chemicals, EDCs, transgenerational, adipogenesis

## Abstract

The incidence of obesity has reached an all-time high, and this increase is observed worldwide. There is a growing need to understand all the factors that contribute to obesity to effectively treat and prevent it and associated comorbidities. The obesogen hypothesis proposes that there are chemicals in our environment termed *obesogens* that can affect individual susceptibility to obesity and thus help explain the recent large increases in obesity. This review discusses current advances in our understanding of how obesogens act to affect health and obesity susceptibility. Newly discovered obesogens and potential obesogens are discussed, together with future directions for research that may help to reduce the impact of these pervasive chemicals.

Obesity is a pandemic that has reached worldwide proportions, affecting essentially every country (1). The most dramatic increase in obesity incidence has occurred over the past 5 decades. Approximately 39.6% of US adults were characterized as obese in 2016, compared to 13.4% in 1980 (2). Even more alarming, the incidence of obesity in children has also been increasing, with 18.5% of US children being characterized as obese in 2016, compared to just 4% before 1980 (2). In some countries, the prevalence of childhood obesity exceeds that of adults (3). Obesity and high body mass index (BMI) are not just cosmetic concerns, but are also associated with comorbidities such as increased risk for heart disease, type 2 diabetes and other metabolic diseases, and cancers, and have contributed to approximately 4 million deaths worldwide between 1980 and 2015 (3). There is an urgent need to understand all the factors contributing to obesity to best implement effective prevention and treatment approaches that have so far proved elusive.

## The Obesogen Hypothesis

The predominant medical explanation continues to be that obesity is the result of a simple imbalance between excessive calorie intake and insufficient energy expenditure—the energy balance or “calories in, calories out” model. However, recent studies have demonstrated that this simple paradigm cannot explain the increase in BMI seen in recent years. A study analyzing National Health and Nutrition Examination Survey (NHANES) data compared BMI between US adults in 1988 and 2006 and found a 2.3-kg/m^2^ increase in adult BMI in 2006 compared with 1988, even at the same amount of caloric intake and energy expenditure (4). Moreover, the quality of carbohydrate calories consumed (high vs low glycemic load) appears to be more important than the total quantity of calories consumed (5, 6). Genetics is widely believed to be associated with obesity; however, known gene variants can explain only 2.7% of the individual variation in BMI (7). Therefore, the 2 most commonly given explanations—genetics and energy balance—cannot fully explain the substantial increases in obesity incidence observed worldwide.

Multiple environmental factors can affect obesity susceptibility (reviewed in 8, 9). These include the gut microbiome composition (10, 11), stress (12), and disrupted circadian rhythms (13), to name a few. Environmental stressors experienced during fetal development have significant impacts on obesity susceptibility later in life. For example, mothers who were in their first and second trimester of pregnancy during the Dutch Hunger Winter of 1944 to 1945 gave birth to children who were predisposed to obesity later in life compared to mothers not exposed to famine during pregnancy (14). Maternal smoking during pregnancy was also shown to lead to a predisposition toward obesity later in life for prenatally exposed children (reviewed in 15).

In 2003, Jerry Heindel put endocrine-disrupting chemicals (EDCs) and obesity on the same map for the first time (16). His idea followed from a proposal originally made by Paula Baillie-Hamilton that increased chemical usage since World War II was responsible for the rapid increase in obesity over the same time period (17). Although it was not justifiable to link increased chemical use, per se, to obesity, Heindel’s proposal that EDCs might be influencing obesity was reasonable because nearly every aspect of the control of appetite, satiety, metabolism, and fat storage is regulated by the endocrine system. Heindel’s proposal eventually ignited research in the area of obesity among researchers already working on EDCs (reviewed in 18).

The idea of EDCs as factors in obesity did not crystallize until it was recognized that certain EDCs could activate nuclear hormone receptors important for the development of white adipocytes, such as peroxisome proliferator–activated receptor γ (PPARγ) (19). Further support came from the findings that EDCs such as tributyltin (TBT) could lead to increased adipogenesis in cell culture models (20-22), bind to and activate PPARγ and its heterodimeric partner, the 9-cis retinoid X receptor (RXR), in vitro (21, 22), and lead to increased adiposity in vivo (22). The identification of chemicals that activated PPARγ to promote adipocyte differentiation and white adipose tissue (WAT) accumulation led to the coining of the term *obesogen* (23).

Obesogens were defined functionally as chemicals that lead to increased WAT accumulation, in vivo, after exposure. The environmental obesogen hypothesis holds that obesogen exposure is an under-recognized and understudied factor in the obesity pandemic (reviewed in 23, 24). Although the obesogen hypothesis was initially controversial, many chemicals known to be obesogenic in animal models are also associated with increased obesity prevalence, BMI, and body weight in humans (9). Research in this area has burgeoned and numerous recent reviews have summarized aspects of obesogen research (eg, 8, 18, 25, 26). It was subsequently recognized that obesogens may have more diverse effects on metabolism than just contributing to obesity; although, obesity may be a key contributor to such effects. These include type 2 diabetes, nonalcoholic fatty liver disease, and the central control of metabolism. Thus, it can be argued that most obesogens are a subset of a larger class of chemicals termed metabolism-disrupting chemicals, not all of which are obesogens (reviewed in 9, 27). Here we summarize what is known about the mechanisms underlying obesogen action, discuss newly identified obesogens and potentially obesogenic chemicals and propose important areas for future research.

## Classic Effects and Mechanisms of Obesogens

**Figure 1.**
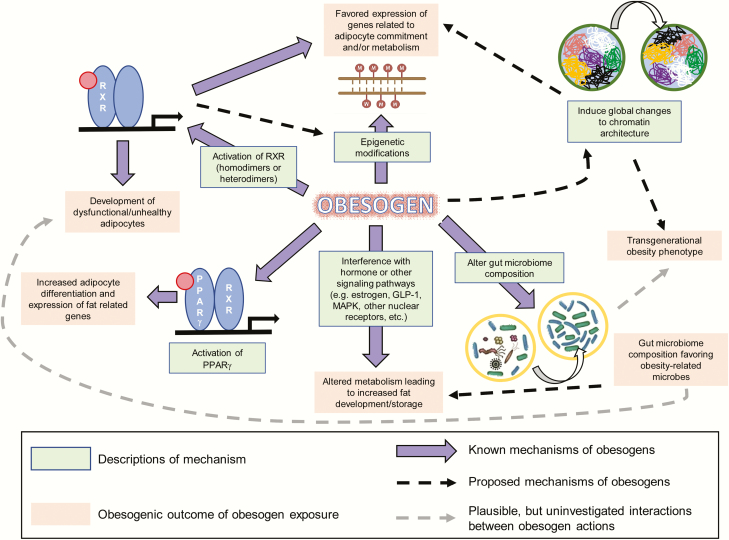
Diagram of known and proposed mechanisms and effects of obesogen exposure. Known mechanisms through which obesogens act are demonstrated by solid purple arrows. Proposed mechanisms are shown by arrows with a dashed black line. Plausible, but uninvestigated, interactions between obesogen actions are shown by arrows with a dashed gray line. Known mechanisms are described in green boxes, and the outcomes of obesogen exposures are shown in pale red boxes.

Obesogens affect the differentiation of white adipocytes, in vitro, and the storage of fat, in vivo, in multiple model organisms (reviewed in 8, 9)(Fig. 1). We distinguish bona fide obesogens that induce increased WAT weight, in vivo, from potential obesogens that can induce adipogenesis, in vitro, but have not yet been demonstrated to induce WAT accumulation in vivo. Numerous potentially obesogenic compounds have been identified using in vitro assays that assess the ability of candidate chemicals to promote differentiation of established cell lines such as 3T3-L1 preadipocytes (28, 29) or primary mouse and human multipotent mesenchymal stromal stem cells (also known as *mesenchymal stem cells* or *MSCs*) into mature adipocytes (29, 30). Many chemicals that promote differentiation of white adipocytes in these assays activate PPARγ and/or RXR (29, 30). This is not surprising because the PPARγ:RXR heterodimer is considered to be the “master regulator of adipogenesis” (31).

One of the most well-characterized obesogens is the organotin, TBT. Organotins are widely used in industry and to some extent in agriculture. Human exposure to organotins can occur via the diet such as seafood contaminated by TBT used in marine shipping applications (32), or as fungicides for paper mills and industrial water systems (reviewed in 33). Triphenyltin use as a fungicide and miticide on high-value food crops presents more opportunities for human exposure (34). TBT contaminates polyvinyl chloride plastics, and organotins are found in samples of house dust (35, 36).

TBT binds to and activates PPARγ and RXR at environmentally relevant (nanomolar; nM) levels, promoting adipogenesis and lipid accumulation (21, 22, 37). Human and mouse MSCs and 3T3-L1 preadipocytes were induced to differentiate into white adipocytes via a PPARγ-dependent pathway after exposure to nM levels of TBT (38, 39). Prenatal TBT exposure diverted bone marrow–derived MSCs preferentially toward the adipose lineage and away from the bone lineage in exposed mice (39) and in mouse MSCs, in vitro (40).

Prenatal and/or perinatal exposure to TBT in mice led to increased body fat in the offspring (22, 41, 42). TBT also induced obesity in mice in both sexes when treated at any age (43-46). Similar effects were observed in rats (46), goldfish (47), and zebrafish (48, 49). Therefore, the obesogenic effects of TBT exposure, developmentally and in adulthood, are well supported in the literature across model systems.

Epidemiological studies of TBT exposures and effects are scant. A longitudinal Finnish cohort study positively associated placental TBT levels with infant weight gain, an established risk factor for adult obesity (50). A recent analysis of NHANES data revealed a strong link between elevated urinary total tin levels and diabetes (51). Human exposure to tin is ubiquitous (52), and it was just shown that plastic specimen containers strongly bind organotins (particularly TBT), sharply impairing recovery (53). Therefore, previous studies of organotin levels in human specimens (eg, 54) probably substantially underestimated TBT levels because of their use of plastic containers during processing and analysis.

A recent clinical study that strongly supports the obesogen hypothesis found that people with the highest blood levels of perfluorinated chemicals had lower resting metabolic rates and regained weight faster after dieting than those with the lowest levels (55). In agreement with predictions from rodent studies (39, 40), the same group showed that humans with the highest blood levels of perfluoroalkyl compounds had lower bone mineral density at baseline and lost bone mineral density faster in a weight-loss trial (56).

Similar obesogenic effects have been observed with other environmental chemicals, such as phthalates, persistent organic pollutants, and components of plastics and epoxy resins. The phthalate MEHP (mono-2-ethylhexyl phthalate) induced adipogenesis in 3T3-L1 preadipocytes via activation of PPARγ (57). DEHP (di-2-ethylhexyl phthalate) induced expression of adipogenic genes in vivo and an obesity phenotype in mice following perinatal exposure (58). Prenatal exposure to bisphenol A (BPA) has been linked to various adverse health effects including reproductive and behavioral issues (59), fat gain in mice and rats (60, 61) and other metabolic outcomes such as type 2 diabetes (62, 63). Some data support the possibility that BPA influences adipogenesis as a PPARγ agonist, although BPA is a relatively weak activator of PPARγ in vitro (64-66). Others have proposed that BPA may induce its obesogenic effects indirectly via its ability to bind to estrogen receptors and interfere with estrogen signaling (67).

Effects of obesogens acting through other nuclear receptors such as the glucocorticoid receptor, estrogen receptors, and androgen receptors have been reported and discussed in detail elsewhere (reviewed in 9, 26). More recently, it has been observed that chronic 52-week exposure of male mice to a mixture of 6 pesticides commonly used in France, at doses equivalent to the tolerable daily intake of each pesticide, led to increased body weight, WAT weight, and glucose intolerance (68). In contrast, similarly exposed female mice exhibited elevated fasting glucose, increased ratio of reduced to oxidized glutathione in the liver, and perturbed levels of microbiome-related urinary metabolites. Loss of the xenobiotic receptor constitutive androstane receptor (CAR) prevented body weight gain and changes in glucose metabolism in male mice, whereas females exhibited increased toxicity, higher body weights, and elevated mortality rates in the absence of CAR (68).

Many obesogens that have been identified using in vivo studies act through nuclear hormone receptor–dependent mechanisms, as do many of those detected using in vitro adipogenesis assays (reviewed in 69). In addition, interaction between nuclear receptors and cross-talk between signaling pathways has been reported (70). However, some of the effects observed in vitro (29) and in vivo, especially transgenerational effects, (25) have not been linked directly to the activation of particular nuclear hormone receptors. Therefore, the mechanisms through which these compounds act appear to be more complex.

## Mechanisms of Obesogen Action: Beyond Peroxisome Proliferator–Activated Receptor γ

Because many known obesogens activate PPARγ and induce adipogenesis, PPARγ activation is widely believed to be a major mechanism through which obesogens can contribute to obesity. PPARγ continues to be the receptor most commonly targeted in screening assays for obesogens (28, 71, 72). However, recent studies have demonstrated alternative and novel mechanisms of obesogen action. These include activation of RXR to induce adipocyte lineage commitment and impair adipocyte health (73, 74), activation of multiple other nuclear receptors, induction of epigenetic modifications in fat tissue (75), alteration of chromatin accessibility or architecture (41, 76), and induction of gut microbiome dysbiosis (77-79). Thus, obesogens have a broad and diverse spectrum of actions. This section discusses some PPARγ-independent mechanisms of obesogens action. A summary of the known and proposed mechanisms through which obesogens can act, together with their possible effects, is illustrated in Fig. 1.

### Adipocyte commitment

TBT was shown to bind to and activate RXR, which is an obligate heterodimeric partner for many nuclear receptors, including PPARγ (80). TBT, as well as selective RXR activators (rexinoids), could commit female mouse MSCs to the adipocyte lineage by activating RXR, whereas selective pharmacological activation of PPARγ could not (74). It was proposed that RXR activation in MSCs inhibits the expression of enhancer of zeste 2, the catalytic subunit of polycomb repressor complex 2, which deposits the repressive histone 3 lysine 27 trimethylation mark (H3K27^me3^) on chromatin. Loss of H3K27^me3^ in TBT-treated MSCs near to genes important for adipogenic commitment led to increased expression of these genes, which largely explains the increased commitment of these MSCs toward the adipose lineage (74). These results also demonstrated that TBT activation of RXR can induce epigenomic modifications, revealing a new mode of action for this well-known toxicant.

### Adipocyte health

Adipocytes have important functions in the maintenance of metabolic health, including glucose and triglyceride uptake from the bloodstream in response to insulin. Disruption of adipocyte function contributes to insulin resistance and type 2 diabetes. Regnier and colleagues first showed that adipocytes induced to differentiate from 3T3-L1 cells by TBT were functionally distinct from adipocytes induced by the PPARγ activator, troglitazone (81). It is generally accepted that selective PPARγ activators, such as troglitazone and rosiglitazone, promote the development of healthy white adipocytes in vitro and in vivo (82, 83). Healthy white adipocytes are characterized by sensitivity to insulin; production of insulin sensitizing, anti-inflammatory adipokines such as adiponectin, apelin, and fibroblast growth factor 21; normoxia; and low to no expression of inflammatory or fibrotic marker genes. Healthy white adipocytes have the ability to undergo conversion to beige adipocytes when treated with thyroid hormone, cold exposure, or β3-adrenergic receptor agonists. These thermogenic adipocytes express genes such as *Ucp1* to uncouple oxidative phosphorylation from adenosine 5′-triphosphate production, leading to the production of heat (73). Activation of RXR by TBT and other rexinoids not only led to increased adipogenic commitment, but the adipocytes treated with TBT or rexinoids during differentiation from MSCs were dysfunctional (73). These adipocytes displayed impaired glucose uptake and insulin signaling, increased expression of inflammatory and fibrotic markers, decreased respiratory function, and reduced expression of beige/brown adipocyte marker genes such as *Ucp1*, *Elovl3*, *Cidea*, and *PPARα* when stimulated with thyroid hormone (73). TBT also failed to induce marker genes characteristic of thermogenic beige/brite adipocytes in a transcriptomal analysis of bone marrow–derived MSCs (84). There is also some evidence that other obesogens can impair thermogenesis in vivo. For example, perinatal exposure to the obesogen DDT led to impaired thermogenesis in the brown adipose tissue, resulting in lower core body temperature and increased susceptibility to high-fat diet–induced insulin resistance in adult female rats (85). Together, these results revealed a new mechanism for obesogen action independent of PPARγ activation. They also demonstrated that some obesogen-induced adipocytes may be dysfunctional, compounding the negative effects of promoting increased accumulation of white fat. Additional in vivo studies will be needed to ascertain the degree to which obesogen exposure alters adipocyte health and how this influences the predisposition to obesity-related diseases.

### Gut microbiome dysbiosis

A relatively unexplored mechanism through which obesogen exposure might predispose exposed individuals to obesity is via alterations to the gut microbiome. It is well established that obesity is associated with composition of the gut microbiome (86, 87). Transplant of the gut microbiome from obese individuals can induce obesity in germ-free mice (11). In contrast, transplant of the microbiome from a lean individual promoted a lean phenotype in similar experiments (11). Transplant of the microbiome from lean donors improved metabolic end points in recipients with metabolic syndrome (11, 88).

Many xenobiotics, including known obesogens, induced changes to gut microbiome composition (77, 89-91). For example, BPA exposure led to increased prevalence of Proteobacteria and Helicobacteraceae together with reduced Clostridia in the gut microbiota of exposed mice, but the study did not investigate whether the altered microbiome was directly associated with any metabolic end points (89). TBT exposure was associated with changes to the gut microbiome in mice, including increased prevalence of Proteobacteria and Helicobaacteraceae, and decreased prevalence of Clostridia, Bifidobacteriaceae, and lactobicillaceae (77). Exposure to triphenyl phosphate increased abundance of Erysipelotrichia and Bacilli and also decreased the prevalence of Clostridia (79). Gut microbial dysbiosis in mice following exposure to triphenyl phosphate (79) or TBT (77) was associated with increased fat accumulation or altered lipid metabolism. However, these studies did not distinguish whether observed differences in the microbiome were the result of metabolic differences in the mice or if the altered microbiome induced the metabolic changes. Monitoring the microbiomes of the dams before and after exposure could help in differentiating between these possibilities, as would conducting a microbiome transplant into germ-free mice. Also, microbial metabolites have been identified as agonistic ligands for the aryl hydrocarbon receptor (AhR) (reviewed in 92). Promoting AhR activity inhibited adipogenesis (93), obesity, and fatty liver both in male and female mice (94, 95) and protected from the effects of a high-fat diet (96). Inhibiting the expression or action of AhR promoted obesity and fatty liver (reviewed in 97).

The “Western dietary pattern” is strongly associated with obesity, and several recent studies have demonstrated that components of the Western diet (eg, ultra-processed food, food additives, artificial sweeteners) can disrupt the gut microbiome (98). Two common dietary emulsifiers, carboxylmethylcellulose and P-80, were recently shown to induce intestinal inflammation and disrupt the gut microbiome, leading to metabolic syndrome in mice together with increased body weight and WAT depot weight (99). (See also “Surfactants” and “Food additives” sections for further discussion on similar emulsifiers and food additives.) Taken together, these results suggest that disrupting the composition of the gut microbiome could be a new mechanism through which obesogen exposure can promote obesity. They also reveal common food additives as a new class of candidate obesogens that require further investigation.

## Possible Mechanisms Underlying Transgenerational Effects of Obesogen Exposure

An intriguing result is that the effects of early-life obesogen exposure can be transmitted to future generations. When pregnant F0 mouse dams were treated with TBT, F1 animals were exposed as embryos, and F2 were exposed as germ cells within F1. F3 and subsequent generations were not exposed; effects in these generations are considered to be transgenerational and permanent (100, 101). It was shown that the effects of TBT treatment on obesity were transgenerational and could be detected in the F1, F2, F3, and F4 descendants of F0 mice exposed during pregnancy (42) or during pregnancy and lactation (41). Interestingly, transgenerational obesity was not observed in similar experiments using the strong PPARγ activator rosiglitazone; therefore, pathways in addition to PPARγ may be required to produce the transgenerational phenotype (42). Alternatively, it might be possible that the “unhealthy” adipocytes produced by TBT exposure are responsible for the transgenerational phenotypes in adipose tissue; this is a ripe area for future study. It was proposed that obesogen exposure can permanently reprogram MSCs to favor the adipose lineage (39). Gene expression in MSCs taken from F1 to F3 generation mice after F0 exposure throughout pregnancy was also biased toward the adipogenic lineage (42). It was suggested that TBT exposure promoted epigenomic changes favoring the development of obesity (41); thus, this may be an example of a maternal programming event leading to a life-long phenotype.

In addition to TBT, heritable effects of several environmental chemicals on obesity have been demonstrated, albeit at relatively high doses. Plastic components such as BPA, diethylhexyl and dibutyl phthalates (58), the pesticide methoxychlor (102), a mixed hydrocarbon mixture (jet fuel JP-8) (103), and the once widely used pesticide, DDT (104) all induced transgenerational obesity in rats, observed in the F3 and/or F4 offspring of following ancestral prenatal or perinatal obesogen exposure to the F0 dams (58, 101-104). The mechanisms underlying these transgenerational effects remain unclear. Some proposed mechanisms for transgenerational effects of obesogen exposure are discussed in this section and illustrated in Fig. 1. Transgenerational effects of obesogen exposure are particularly concerning because current risk assessment paradigms do not consider this “generational toxicology” (105).

### Epigenetic modifications

Epigenetic modifications to the genome are one mechanism through which environmental factors, such as chemical exposure, can alter gene expression and lead to adverse outcomes. Many epigenetic modifications have been observed after obesogen exposure. For example, the obesogen TBT induced global changes to DNA methylation (106) and to histone methylation (74) in vitro. Many environmental chemicals, including known obesogens, led to epigenetic modifications in vivo and altered epigenetic signatures and obesity phenotypes in unexposed generations (58, 75, 103, 104). It has been suggested that such epimutations can be inherited (58, 104, 107) and that chemical exposures during fetal development can induce epigenetic changes in the germline leading to observed phenotypes in subsequent generations (108).

Some argue that altered DNA methylation itself may be transmitted across generations (109, 110), but others contest these results and hold that DNA methylation is not readily heritable (111, 112). This is because the zygote undergoes genome-wide erasure of epigenetic marks shortly after fertilization, and the developing germ cells experience an additional wave of global demethylation as they mature (113-115). These stages of global demethylation both in the zygote genome and in the developing germline would likely prevent inheritance of most altered DNA methylation marks. There is some discussion about some DNA methylation escaping reprogramming in an “imprinting-like” mechanism; however, the presence of DNA methylation at particular sites has not been demonstrated across generations. Indeed, the opposite has been shown; there is little, if any consistency in DNA methylation across generations and among tissues in transgenerational experiments (75, 105, 116, 117). The consensus from a variety of exposures is that altered DNA methylation can be detected in the F3 generation and beyond in transgenerational experiments, but a plausible mechanism for how these changes are transmitted across generations is lacking.

In addition to DNA methylation, other types of epigenetic changes have been observed following ancestral chemical exposure. Histone modifications are capable of inducing changes to chromatin packaging and therefore, DNA accessibility and gene expression. In the male germline, a majority of histones are removed during spermatogenesis. However, 5% to 15% of histones are retained in mammalian sperm. Analysis of the sperm of F3 rats ancestrally exposed to the pesticide DDT or the fungicide vinclozolin revealed additional differential histone retention sites for histone H3 compared to the sperm of control lineage F3 animals (118). Histone retention in the sperm can be affected by environmental toxicant exposure and might be transmitted to unexposed generations. However, the underlying mechanisms and the importance of these retention sites on the development of disease phenotypes require further investigation. Noncoding RNA (ncRNA) expression is another type of epigenetic change that has been observed in transgenerational experiments. More than 200 differentially expressed small noncoding RNAs (sncRNAs) were identified in the sperm between F3 rats ancestrally exposed to vinclozolin compared with controls (119). Some of these dysregulated sncRNAs correlated with messenger RNA profiles observed in diseased tissues in animals ancestrally exposed to vinclozolin. Although differences both in ncRNA and differential histone retention sites have been observed in transgenerational experiments after exposure to environmental toxicants, these changes have not been linked mechanistically to transgenerational obesity.

### Chromatin accessibility

One potential explanation for how epigenomic changes are observed in unexposed generations was recently proposed (41, 76). In this model, TBT exposure induced global changes to chromatin organization that resulted in changes in DNA methylation. Epigenetic analysis of the fat tissue of obese F4 male mice ancestrally exposed to the obesogen TBT revealed more than 10 000 regions where methylation was altered in 4 out of 4 animals from the TBT group vs controls. However, none of these regions was closely associated with the promoters of genes whose expression was altered in these animals. When methylation was analyzed in a different way, assessing larger blocks of differentially methylated regions in genomic DNA with the same direction of methylation, the result was different (41). These regions were called isodirectionally differentially methylated blocks, or isoDMBs. Hypomethylated isoDMBs were associated with overexpression of metabolism related genes, such as leptin, in fat tissue of F4 mice ancestrally exposed to TBT (41). Investigation into chromatin accessibility of the sperm of F3 or F4 mice revealed that the hypomethylated isoDMBs in the WAT coincided with decreased chromatin accessibility in the same regions of the sperm (41). It was proposed that TBT exposure altered chromatin architecture, which resulted in decreased chromatin accessibility of regions where genes important for metabolism were located, producing a “thrifty phenotype” (41).

A deeper analysis of liver, MSCs, and WAT from the same experiment revealed that the transcriptomes of tissues of mice ancestrally exposed to TBT showed a bias in the expression of genes related to chromatin organization, chromosome organization, and metabolic processes compared with the control group and that these differences spanned generations (F3, F4), tissue types (liver, MSCs, WAT), and ontogeny (mesoderm vs endoderm) (76). The authors inferred that TBT exposure disrupted chromatin and chromosome organization and that this disrupted structure was able to self-reconstruct in subsequent generations, much as the normal structure is able to do in controls (76). In this model, the disrupted chromatin structure leads to alterations in DNA methylation, histone retention, and the expression of messenger RNAs as well as ncRNAs rather than these epigenomic alterations being inherited directly (76). However, the molecular mechanisms underlying this disrupted structure and how it might self-reconstruct in subsequent generations remain unknown at present.

## Newly Discovered Obesogens (New Threats)

Obesogens have been extensively reviewed in recent years and several publications report and categorize known obesogens (reviewed in 8, 26, 69). Additional chemicals are being identified that may act as obesogens in vitro and in vivo. This section summarizes recent findings concerning new potential obesogens and how their action (when known) compares with that of model obesogens, such as TBT.

### Dibutyltin

Dibutyltin (DBT) is the major breakdown product of TBT in vivo and is more prevalent in the environment than TBT because of its presence at substantial concentrations (up to 3% w/w) in polyvinyl chloride (PVC) plastics (120). DBT has been demonstrated to leach into drinking water from PVC pipes and, therefore, may produce a hazard to humans (121). DBT activated the same receptors as does TBT and induced 3T3-L1 preadipocytes (122) and human and mouse MSCs to differentiate into adipocytes (123). In addition, perinatal exposure to DBT led to increased WAT weight in mice comparable to that of the model obesogen TBT, although a higher dose of DBT was required to achieve the same effect as TBT (123). Surprisingly, offspring of DBT-exposed dams demonstrated insulin resistance, a phenotype not observed with TBT exposure (123). This raises the possibility that DBT may engage additional cellular mechanisms to those through which TBT acts. The effects of DBT on other phenotypes elicited by TBT, such as reprogramming stem cell fate, fatty liver, impairing thermogenesis, and transgenerational transmission have not yet been investigated.

### Bisphenol A analogues

Considerable evidence on the adverse health effects of BPA exposure has led to efforts to produce BPA-free plastics. However, related chemicals such as bisphenol S (BPS) and bisphenol F (BPF), are often used to replace BPA in these new plastics as companies strive to develop BPA-free products but retain current manufacturing processes (124). The toxicity of these BPA analogues is less well understood. Some studies have demonstrated that these bisphenols also have endocrine disrupting properties similar to BPA (reviewed in 125). Interestingly, BPS and halogenated BPA analogues demonstrated higher activation of PPARγ and potency in inducing adipogenesis than did BPA (64, 65). A recent study revealed that perinatal exposure to BPS elicited obesity in mice (126). Although exposure levels of BPA have been significantly associated with obesity incidence, levels for BPS and BPF were not linked with obesity in a cross-sectional study of adults after adjusting for lifestyle and socioeconomic factors (124). Interestingly, a newer longitudinal birth cohort study revealed that BPS and BPF were significantly associated with obesity in children (age 6-19), whereas BPA and total bisphenol levels were not significantly associated (127). These results suggest substituting other bisphenols for BPA may not be an effective strategy for mitigating the hazards of BPA to humans.

### Acrylamide

Acrylamide is a chemical widely used in the manufacture of paper, dye, and other industrial products. It can also be formed as an unintentional byproduct of cooking carbohydrate-containing foods at high temperatures by frying, baking, or roasting, which is probably the main source of human exposure (128). A recent study reported that acrylamide exposure induced fat accumulation in male mice when fed a high-fat diet (129). Although acrylamide apparently increased PPARγ expression, it was not identified as a PPARγ activator. Instead, it was that acrylamide acted through the mitogen-activated protein kinasd and AMPK-ACC (adenosine 5′-monophosphate–activated protein kinase–acetyl-CoA carboxylase) pathways (129). Studies in 2 different longitudinal birth cohort studies from (France) (130) and Norway (131) demonstrated that children prenatally exposed to higher levels of acrylamide were more likely to be born small for gestational age and obese at age 3 years. Hemoglobin adducts of acrylamide (HbAA) and glycidamide (HbGA) were proposed as biomarkers of acrylamide exposure in humans. One analysis of NHANES data (2003-2006) demonstrated a positive association between HbGA levels and obesity, but a negative association between HbAA and obesity (132). In contrast, another analysis of NHANES data (2003-2004) found a negative association with obesity for HbAA and no association with HbGA (133). Clearly more data are required to establish whether acrylamide exposure is linked with obesity, but the ubiquitous exposure of the population to acrylamide from baked and fried foods indicates that such data will be very important.

### Surfactants

Dioctyl sodium sulfosuccinate (DOSS) is commonly used as a dietary emulsifier and as a major component of an over-the-counter and commonly recommended stool softener (Colace/Docusate). DOSS was also a principal component in the COREXIT (Corexit Environmental Solutions LLC, Nalco Holding Company) dispersants that were used in the cleanup of the Deepwater Horizon oil spill in 2010 (134). DOSS activated PPARγ and induced adipogenesis in vitro (135). A recent in vivo mouse study showed that perinatal exposure of pregnant mouse dams to DOSS led to obesity in their offspring (136). A second commonly used surfactant (and component of COREXIT), Span-80 (sorbitan monooleate, Croda International PLC.), activated RXRα and induced 3T3-L1 preadipocytes to differentiate into adipocytes (137). When 3T3-L1 cells were treated with a combination of Span-80 and DOSS, adipogenic induction was greater than with either chemical individually (137). These results establish surfactants as an unexplored category of obesogens requiring further investigation. Because both of these chemicals are commonly used as food additives, it will also be interesting to test whether they affect the gut microbiome to induce obesity phenotypes in vivo, as other dietary emulsifiers have been shown to do (99).

### Food additives

Increasing evidence has emerged linking components of the “Western dietary pattern” (eg, ultra-processed, food additives) to obesity. Notably, commonly used food additives have been shown to have obesogenic potential. The dietary emulsifiers carboxymethylcelluclose and P-80 (see “Gut Microbiome Dysbiosis” section) as well as DOSS and Span-80 (see “Surfactants” section) induced adipogenesis in vitro and/or in vivo. Other food additives have also been shown to have obesogenic potential. The widely used food preservative 3-tert-butyl-4-hydroxyanisole (3-BHA) induced adipocyte differentiation in 3T3-L1 preadipocytes (138), and 3-BHA exposure increased adiposity and lipid plasma levels in exposed mice (139). The flavor enhancer MSG (monosodium glutamate) was long ago shown to be an obesogen in vivo (140). Two studies showed that MSG may induce its adipogenic effects by impairing secretion of glucagon-like peptide-1, an important hormone regulating appetite and satiety (141) and/or by antagonizing androgen receptor action (142). Taken together, these studies provide insight into why the consumption of highly processed food leads to greater weight gain than consumption of the same number of calories from fresh foods (5, 6). This is a ripe area for future investigation.

### Pesticides

Many pesticides or their major breakdown products have been linked to obesity in animals and in humans (reviewed in 143). One example is DDT in rats (104) and humans (144) and its major breakdown product DDE in humans (145). Although DDT was banned under the Stockholm Convention, it persists in the environment and continues to be used in some countries (particularly in Africa). Methoxychlor induced obesity in rats (102), and other pesticides have been identified as candidate obesogens. The widely used, neonicotinoid insecticide, imidacloprid, was recently shown to induce 3T3-L1 preadipocytes to differentiate into adipocytes (as measured by lipid accumulation and marker gene expression) (146) and to promote high-fat diet–induced obesity in mice (147). Glyphosate, the most highly used herbicide worldwide, induced obesity in F2 and F3 offspring of F0 female rats exposed during gestation (148). The herbicide quizalofop-p-ethyl induced adipogenesis in 3T3-L1 preadipocytes (149). A variety of other agrochemicals induced adipogenesis in 3T3-L1 preadipocytes and in mouse and human MSCs (29, 30). Although the potential of many of these agrochemicals to promote obesity in vivo has not yet been explored, the intensive use of such chemicals and the widespread human exposure via consumption of foods indicates that such studies will be important to understand the contributions of agrochemical exposure to obesity.

## Future Directions

Although the environmental obesogen field is just 15 years old, it is becoming clear that chemical exposures may be important contributors to the obesity pandemic. Many advances have been made into potential mechanisms underlying obesogen action and how obesogen exposure may predispose humans and animals to obesity (Fig. 1). However, we have only just scratched the surface and need to learn much more about the number of obesogens that exist, how they act, and how we can best protect ourselves and future generations from their harmful impacts. A combination of mechanistic studies in cell and animal models together with longitudinal epidemiological and biomonitoring studies in humans will be required for a full assessment of the risks and costs of these exposures to public health. Early estimates suggest that these costs may be substantial (150, 151).

Many of the recent discoveries regarding the mechanisms of obesogen action are still in the early stages and require more research to determine their significance in obesity susceptibility. We currently know very little about how obesogen exposure can interact with diet to promote obesity. We know that obesogens can affect composition of the microbiome and that microbiome composition itself can cause obesity. Yet we know little about how obesogen-elicited changes in the microbiome can contribute to obesity or whether observed changes in microbiome composition are the cause or consequence of obesity and obesogen-induced metabolic abnormalities.

Sexually dimorphic effects of obesogens are quite common. The first chemical to be reported as an obesogen in vivo was the synthetic estrogen diethylstilbestrol, which elicited obesity after perinatal exposure only in adult female mice (152). Such effects might be mediated via the estrogen receptors, but this remains to be demonstrated. TBT elicited increased fat mass in both sexes of the F1 generation, but obesity was found only in males of the F2 to F4 generations and it was not possible to link these effects with sex steroid receptors (41, 42, 123). Many other examples of sexually dimorphic effects of obesogen exposure exist in animal models (reviewed in 9). The incidence of obesity is increasing in both sexes in human populations; however, the effect is most striking in female patients, particularly in the United States (2). We currently know very little about the etiology of these sexual dimorphisms beyond some indications that effects of environmental estrogens, such as BPA, may be expected to be more pronounced in girls and women. Because most obesogens do not act via the estrogen receptors, it will be important to understand which pathways mediate obesogen action to formulate appropriate strategies for intervention and prevention.

It will be important to understand the effects of mixtures on obesity and whether combined exposure of obesogens will interact in a positive or synergistic manner, or if they will instead cancel each other’s effects. Many obesogens appear to induce a variety of effects and, therefore, may be acting through multiple mechanisms. TBT, for example, activates PPARγ and RXR, but also induces epigenetic modifications and changes to chromatin architecture (41, 76). However, it is not known how these changes in chromatin architecture can be transmitted to future generations. Multiple chemicals have been demonstrated to elicit transgenerational effects on obesity, but we know virtually nothing about how these effects are carried across generations (reviewed in 69, 105). Nuclear receptor activation can lead to epigenetic alterations (153, 154), but this connection has not yet been characterized as part of the mechanism of action of obesogens. Many studies have established that the microbiome can be linked to epigenetic changes (155, 156), but it remains to be shown whether the effects of obesogen exposure on the microbiome and the epigenome are causally related. It is also possible that the impact of obesogens on the obesity pandemic could be the result of exposure to a combination of obesogens, each of which may act through a different pathway, rather than through a single pathway. Many studies have found that chemical mixtures can induce higher receptor activation or stronger phenotypes (157-160).

Although we have learned quite a bit about the number and nature of obesogens and have gleaned clues to how some act, we know relatively little about the full spectrum of obesogens and their mechanisms of action. Understanding how obesogens act will pave the way for identifying new obesogens that may act through similar mechanisms. What is needed for EDCs in general, and obesogens in particular, are better screening systems that can identify likely candidates for in-depth screening. ToxCast and Tox21 have been touted as the future of such screening studies but they may not be adequate for the task (29). It will be important to establish standardized, internationally harmonized screening methods that are reliable, robust, and perform reproducibly across laboratories. The European Union under its Horizon 2020 program has funded 8 international consortia to develop such screening assays to identify EDCs. Three of these consortia are focused on developing methods to identify metabolism-disrupting chemicals. Such efforts will lay the foundation for international efforts to identify potential EDCs and obesogens. Because some of these chemicals may have multigenerational or transgenerational effects, their identification should be an urgent public health goal.

Lastly, one may wonder to what extent the effects of obesogen exposure can be treated and whether it is possible to avoid exposure to such chemicals. One unfortunate consequence of living in an industrialized society is exposure to a plethora of synthetic chemicals. Despite substantial evidence for the widespread effects of EDCs on laboratory animals, wildlife, and humans, changing public policy to limit exposures has proven to be difficult. No doubt it will be equally difficult to implement public policies designed to reduce exposure to obesogens. It seems to be much more likely that effective changes in public policy will be best implemented at the level of local cities and school boards where the influence of concerned citizens equals or exceeds that of industry lobbyists, compared with the state and federal levels where the opposite prevails. Certainly, this has proven to be the case in Southern California, where a number of cities and school districts have implemented policies to greatly reduce exposure to toxic chemicals, including EDCs. For the foreseeable future, the most effective approach will probably be personal action to reduce or eliminate our exposures. We recommend implementing a personal “precautionary principle” whereby we take steps to eliminate EDCs and obesogens in our own diets, personal care products, and lifestyles to the extent possible. The rise in availability of fresh organically grown foods, personal care products lacking EDCs, and cleaning products lacking toxic ingredients, along with an increase in movements to reduce the use of single-use plastics, for example, is testimony to the powerful effects that our buying choices in the marketplace have on corporate behavior.

## Abbreviations

AhR: aryl hydrocarbon receptor
BMI: body mass index
BPA: bisphenol A
BPF: bisphenol F
BPS: bisphenol S
CAR: constitutive androstane receptor
DBT: dibutyltin
DDT: dichlorodiphenyltrichloroethane
DEHP: di-2-ethylhexyl phthalate
DOSS: dioctyl sulfosuccinate
EDCs: endocrine-disrupting chemicals
HbAA: hemoglobin adducts of acrylamide
HbGA: hemoglobin adducts of glycidamide
isoDMBs: isodirectionally differentially methylated blocks
MEHP: mono-2-ethylhexyl phthalate
MSCs: mesenchymal stem cells
NHANES: National Health and Nutrition Examination Survey
PPARγ: peroxisome proliferator–activated receptor γ
PVC: polyvinyl chloride
RXR: retinoid X receptor
TBT: tributyltin
WAT: white adipose tissue

## Additional Information

***Disclosure Summary:*** B.B. is a named inventor on US patents related to PPARγ and other nuclear receptors, some of which have been licensed to for-profit entities. None of these present any conflict of interest. R.J.E. has nothing to disclose.
